# Prizes and Heroes: Lagging and leading indicators

**DOI:** 10.1186/1742-4690-7-87

**Published:** 2010-10-13

**Authors:** Kuan-Teh Jeang

**Affiliations:** 1 The National Institutes of Health, Bethesda, MD, USA

## Abstract

What are the purposes of prizes and recognitions? Are they lagging indicators of past achievements or leading indicators of things to come?

## 

Every October there is a flurry of Nobel prize announcements (see e.g. [1]). In reflecting on Nobel and other prizes, one can ponder a couple of questions. Why are prizes given? And why are certain recipients chosen preferentially?

From a common sense perspective, prizes seem to be disbursed for two categories of purposes. In the first category, a prize is given to recognize past achievements of high significance. Here, many prize-givers benefit from "gilt by association". By choosing big-name winners, the luster of the awardee adds to the prestige of the prize. Thus, the selection for these prize recipients may subscribe to a sociological "halo" or "Matthew" effect [2] whereby prominent scientists tend to be more favorably evaluated, and these individuals gather more and more peer recognition while less prominent peers tend not to be accorded similar credit for what they do. Not uncommonly, a pattern emerges whereby only a small circle of names garner the lion's share of prizes.

In the second category, prizes are awarded with the intention to motivate future achievements. CNN's Hero of the Year http://www.cnn.com/heroes is an example of this prize category. For example, Efren Peñaflorida of the Philippines was recognized as a 2009 Hero for creating mobile pushcart classrooms. By receiving this recognition, Penaflorida was able to leverage the award to create more visibility and more pushcarts for his program. Thus, this category of prizes rather than being a lagging indicator of past achievement serves a leading predictor of hope for future progress. While most Nobel prizes do highlight past work, the Nobel Peace Prize is frequently given with the intention of galvanizing change, as was the case for last year's award to President Obama.

*Retrovirology *has discussed that equitable means for highlighting scientific achievements are difficult to achieve [3,4]. Each year, *Retrovirology *awards a Prize to recognize a worthy mid-career scientist (e.g. [5,6]). The intention of the *Retrovirology Prize *is to marry the concept of past achievements with the potential for further accomplishments in retrovirus research. As we open the nomination period for the 2010 *Retrovirology Prize*, we are mindful of the need to prospectively evaluate our past winners to see how they may have fulfilled and will fulfill hopes of post-prize achievements.

## Authors' contributions

KTJ wrote this editorial which reflects his personal opinion and does not represent the views of KTJ's employer, the US National Institutes of Health.
